# GAS5 Downregulation Promotes the Progression of Triple-Negative Breast Cancer by Regulating Tumor Cells-MDSCs Crosstalk

**DOI:** 10.3390/cancers18193133

**Published:** 2026-09-27

**Authors:** Junze Du, Xingyun He, Lingfeng Tang, Hao Liu, Qingqiu Chen, Sisi Li, Gang Tu, Shichao Li

**Affiliations:** 1 Chongqing Key Laboratory of Molecular Oncology and Epigenetics, Department of Breast and Thyroid Surgery, The First Affiliated Hospital of Chongqing Medical University, Chongqing 400016, China; dujunze@tmmu.edu.cn (J.D.); tlf1994@hospital.cqmu.edu.cn (L.T.); 2024440508@stu.cqmu.edu.cn (H.L.); 2 Department of Breast and Thyroid Surgery, The First Affiliated Hospital of Army Medical University, Chongqing 400038, China; jcgaqq@tmmu.edu.cn; 3 Department of Radiology, The First Affiliated Hospital of Army Medical University, Chongqing 400038, China; hxy17263@tmmu.edu.cn; 4 The Biobank of Southwest Hospital, The First Affiliated Hospital of Army Medical University, Chongqing 400038, China; chssl@tmmu.edu.cn; 5 Chongqing Key Laboratory of Translational Research for Cancer Metastasis and Individualized Treatment, Chongqing University Cancer Hospital, Chongqing 401300, China

**Keywords:** growth arrest-specific 5 (GAS5), myeloid-derived immunosuppressor cells (MDSCs), triple-negative breast cancer (TNBC), YTHDF2/MYBL2/CCL2 axis, IL-6/STAT3/UPF1 axis

## Abstract

Background: Triple-negative breast cancer (TNBC) is exceptionally aggressive, with a high recurrence rate, resistance to chemotherapy, and a tendency to metastasize. The long non-coding RNA growth arrest-specific 5 (GAS5) is associated with TNBC progression; however, the specific mechanism still requires further exploration. Methods: Integrative bioinformatics analyses and qRT-PCR were used to investigate the significant downregulation of GAS5 in TNBC cells and tissues. The functional role and molecular mechanism of GAS5 in inhibiting the progression of TNBC and increasing sensitivity to chemotherapy were investigated using in vitro experiments, including qPCR, Western blot, CCK8 assay, Transwell, Chemotaxis assays, RNA immunoprecipitation, m6A-RNA immunoprecipitation, Chromatin immunoprecipitation, Flow cytometry, ELISA, and Luciferase reporter assays, and in vivo experiments using tumor xenografts. Results: GAS5 was significantly downregulated in TNBC cells and tissues, as well as in chemotherapy-resistant conditions. Functional assays revealed that overexpression of GAS5 attenuated the progression of TNBC and increased their sensitivity to docetaxel (DTX) both in vitro and in vivo. Furthermore, we confirmed that GAS5 recruited YTHDF2 to target MYBL2, which in turn downregulated the chemokine CCL2, which reduced the recruitment of myeloid-derived immunosuppressive cells (MDSCs). In addition, IL-6 from MDSCs downregulated GAS5 to promote TNBC growth and attenuate the sensitivity of TNBC to DTX through the STAT3/UPF1 axis, forming a feedback loop between tumor cells and MDSCs. Conclusions: These findings illustrated the significance of the GAS5-mediated mutual regulatory pathway between tumor cells and MDSCs in TNBC and provide new avenues for the development of targeted therapies in TNBC.

## Simple Summary

Triple-negative breast cancer (TNBC) is exceptionally aggressive. The long non-coding RNA growth arrest-specific 5 (GAS5) is associated with TNBC progression; however, the specific mechanism still requires further exploration. Herein, we observed a significant downregulation of GAS5 in TNBC cells and tissues, as well as in chemotherapy-resistant conditions. Functional analyses demonstrated that GAS5 overexpression inhibits the progression of TNBC and increases the sensitivity to chemotherapy. Mechanistically, GAS5 repressed MDSC infiltration through the YTHDF2/MYBL2/CCL2 axis, while MDSC-derived IL-6 downregulated GAS5 through STAT3/UPF1 signaling, establishing a feedback loop between tumor cells and MDSCs. These findings illustrate the significance of the GAS5-mediated mutual regulatory pathway between tumor cells and MDSCs in TNBC and provide new avenues for the development of targeted therapies in TNBC.

## 1. Introduction

TNBC is an exceptionally aggressive subtype of breast cancer (BC) [1]. The high metastasis, lack of effective therapeutic targets, and drug resistance have become obstacles in the treatment of TNBC. Hence, it is imperative to unravel the pathogenesis of TNBC to explore effective treatment strategies.

Recent research has demonstrated that the interrelationship between tumor cells and the tumor microenvironment (TME) is involved in regulating the growth, metastasis, immune evasion, and drug resistance of tumors [2]. Myeloid-derived suppressor cells (MDSCs) are a major component of the TME, which play a significant role in promoting immune suppression [3]. It has been reported that an increased number of MDSCs in TNBC patients is associated with poor patient survival [4,5]. MDSCs interact reciprocally with malignant cells through paracrine or extracellular matrix interactions, which jointly participate in the regulation of tumor progression [6,7]. However, the precise mechanisms by which MDSCs interact with tumor cells to accelerate the progression of TNBC are still not fully understood. Understanding the mechanism of crosstalk between TNBC cells and MDSCs represents a promising strategy for treating TNBC that targets both the TME and cancer cells.

GAS5, as a long non-coding RNA, has been reported to be downregulated in multiple cancers and acts as a tumor suppressor via various mechanisms [8]. Previous studies have also found that low GAS5 expression in BC is associated with the clinical characteristics of this cancer, such as metastasis, recurrence, and resistance to chemotherapy [9,10]. Studies show that GAS5 is lowly expressed in TNBC tissues and cells, and is associated with the progression and prognosis of TNBC [11,12]. However, the underlying mechanisms of GAS5 dysregulation and its functions still require further exploration. Previous literature revealed the significant involvement of the interaction between cancer cells and the TME as a pivotal mechanism contributing to tumor progression [6,7]. Therefore, we hypothesize that TNBC with low GAS5 expression may participate in regulating TNBC progression through mutual modulation of MDSCs.

In this study, we observed a significant downregulation of GAS5 in TNBC tissues, correlated with chemoresistance. GAS5 overexpression inhibited the progression of TNBC and increased sensitivity to chemotherapy. Subsequent experiments further elucidated that GAS5 downregulation prominently drove MDSC recruitment via the YTHDF2/MYBL2/CCL2 axis. In turn, IL-6 from infiltrating MDSCs downregulated GAS5 to promote TNBC growth and attenuate the sensitivity of TNBC to docetaxel through the STAT3/UPF1 signaling pathway, establishing a feedback loop between TNBC cells and MDSCs. In summary, a deeper understanding of the function and mechanism of the GAS5-mediated mutual regulatory pathway between tumor cells and MDSCs in regulating the progression of TNBC holds great potential to identify new therapeutic targets for TNBC.

## 2. Materials and Methods

### 2.1. Clinical Specimens

Triple-negative breast cancer and adjacent normal tissues were collected from patients undergoing breast cancer surgery in the Department of Breast and Thyroid Surgery, the First Affiliated Hospital of Army Medical University. Before collecting the samples, written consent was obtained from patients. The application of these tissues was conducted in compliance with ethical guidelines by the Ethics Committee of the First Affiliated Hospital of Army Medical University (No: (B)KY2025083).

### 2.2. Cell Lines and Establishment of Stable Cells

TNBC cell lines MDA-MB-231, HCC1937, MDA-MB-468, and Hs578T were obtained from the American Type Culture Collection (ATCC). Cells were cultured in Dulbecco’s Modified Eagle Medium (DMEM) (Thermo Fisher Scientific, Waltham, MA, USA) supplemented with 10% fetal bovine serum (FBS) (Gibco, USA) and 1% penicillin-streptomycin in a 37 °C incubator with 5% CO_2_. The MCF10A cell, sourced from American Type Culture Collection (ATCC), was cultured in Dulbecco’s Modified Eagle Medium: F-12(DMEM/F-12) (Thermo Fisher Scientific, Waltham, MA, USA) supplemented with 5% horse serum, 50 ng/mL epidermal growth factor (EGF), 10 mg/mL insulin, 0.5 mg/mL hydrocortisone, 0.1 mg/mL cholera toxin, and 1% streptomycin-penicillin in a 37 °C incubator with 5% CO_2_.

As previously described [13], docetaxel (DTX) resistant MDA-MB-231 and HCC1937 cells (MDA-MB-231/DTX and HCC1937/DTX) were generated from sensitive cell lines by culturing them with gradually increasing doses of docetaxel. Initially, sensitive cells were treated with 20 nM DTX for 48 h. Then, dead cells sensitive to DTX were removed, and the live cells were recovered by changing to fresh culture medium for 72 h. After passaging, live cells were again treated with higher concentrations of DTX (40 nM) for 48 h and then cultured in fresh medium for 72 h. Using the above method, we gradually increased the dose of DTX and eventually1000 nM DTX was used to treat the cells. Once cells could proliferate stably in 1000 nM DTX, they were designated as DTX-resistant sublines.

Lentiviral particles carrying shRNA targeting GAS5, STAT3, or overexpression constructs for GAS5 were packaged in HEK293T cells using the pLKO.1-puro (shRNA) or pCDH-CMV-MCS-EF1-puro (overexpression) vector systems with psPAX2 and pMD2.G packaging plasmids. Virus-containing supernatants were harvested at 48 h and 72 h post-transfection, filtered through a 0.45 μm membrane, and concentrated by ultracentrifugation. MDA-MB-231 and HCC1937 cells were infected with lentivirus in the presence of 8 μg/mL polybrene for 24 h. Stable integrants were selected with puromycin (2 μg/mL) for 7–10 days. Surviving colonies were pooled and expanded.

### 2.3. MDSCs Identification and Subset Analysis

For MDSC identification and subset analysis, single cells were stained with fluorochrome-conjugated antibodies against CD11b, CD33, HLA-DR, CD14, and CD15 (antibodies listed in Supplementary Table S3). Total MDSCs were defined as CD11b^+^CD33^+^HLA-DR^−^/low. Monocytic MDSCs (M-MDSCs) were defined as CD11b^+^CD33^+^HLA-DR^−^/lowCD14^+^CD15^−^ and polymorphonuclear MDSCs (PMN-MDSCs) were defined as CD11b^+^CD33^+^HLA-DR^−^/lowCD14^−^CD15^+^, according to established guidelines [14]. MDSCs were isolated from two sources: (1) healthy donor PBMCs (*n* = 5 independent donors) for chemotaxis assays, and (2) primary TNBC tissues (*n* = 6 independent patients) for co-culture experiments. Isolation was performed by fluorescence-activated cell sorting (FACS) using a BD FACSAria III. Post-sort purity was consistently >95%, and viability was >90% for PBMC-derived MDSCs and >85% for tumor-derived MDSCs, as determined by Trypan blue exclusion. Each experiment was performed using MDSCs from at least three independent donors or patients, and the data shown are representative of at least three independent experiments.

To functionally validate the immunosuppressive capacity of isolated MDSC subsets, autologous CD3^+^T cells were purified from healthy donor PBMCs by positive selection using CD3 microbeads (Miltenyi Biotec, Shanghai, China) according to the manufacturer’s instructions. Purified CD3^+^T cells were then stimulated with anti-CD3/CD28 Dynabeads (Thermo Fisher Scientific, Waltham, MA, USA) at a bead-to-cell ratio of 1:1 in complete RPMI 1640 medium supplemented with 10% FBS, 100 U/mL penicillin, 100 μg/mL streptomycin, and 20 U/mL human IL-2 (PeproTech, Suzhou, China). Sorted M-MDSCs and PMN-MDSCs were added to the stimulated T cells at a 1:1 MDSC-to-T-cell ratio. As controls, T cells were cultured alone without MDSCs. After 72 h of co-culture at 37 °C with 5% CO_2_, cells were harvested and stained with anti-BrdU. BrdU dilution in live CD3^+^T cells was analyzed on a BD FACSAria III flow cytometer using FlowJo software (v10.8). Each assay was performed in triplicate using MDSCs from at least three independent donors (Supplementary Figure S1).

### 2.4. Plasmids and Transfection

The full-length GAS5 sequence was synthesized by Sangon Biotech Co., Ltd. (Shanghai, China) and cloned into lentiviral vector pCDH-CMV-MCS-EF1-puro to construct a GAS5 overexpression plasmid, which was then used to infect cells to generate stable cells overexpressing GAS5. For GAS5 knockdown, shGAS5 was synthesized by Sangon Biotech Co., Ltd. (Shanghai, China) and cloned into a pLKO.1-puro lentiviral vector to construct the pLKO.1-shGAS5 plasmid. The coding sequences of human YTHDF2, MYBL2, and UPF1 were cloned into the pcDNA3.1 plasmid, which was named YTHDF2-OE, MYBL2-OE, and UPF1-OE (Sangon Biotech, Shanghai, China). For luciferase reporter assay, the human CCL2 promoter (2.1 kb) containing the wild type (WT) MYBL2 binding site (5′-GGCTATTTAACCCTT-3′) or mutant (Mut) MYBL2 binding site (5′-GGACGCCGGTATGCTT-3′, the mutated bases were underlined), or the human UPF1 promoter (2.1 kb) containing the wild type STAT3 binding site (5′-TTCCAAGCA-3′) or mutant STAT3 binding site (5′-TGATGCATA-3′) was separately cloned into pGL3-Basic luciferase vector (Promega, Madison, WI, USA) to construct the pGL3-MYBL2-WT, pGL3-MYBL2-Mut, pGL3-UPF1-WT, and pGL3-UPF1-Mut plasmids. Using the SRAMP database, we identified two high-confidence DRACH motifs (GGACA and TGACC) within the MYBL2 mRNA as candidate m6A sites. According to the predicted m6A sites, the dual-luciferase reporter containing wild-type m6A sites (MYBL2-WT) or mutant m6A sites (GTGAA and TACTC; the mutated bases were underlined) was synthesized and subcloned into the pmirGlo luciferase vector by Sangon Biotech Co., Ltd. (Shanghai, China). TNBC cells were transfected with plasmids utilizing Lipofectamine 3000 reagent (ThermoFisher) according to the manufacturer’s instructions. Transfected cells were harvested for subsequent evaluation.

### 2.5. Quantitative Reverse Transcription Polymerase Chain Reaction

Total RNA was extracted from tissues or cells with TRIzol reagent (Thermo Fisher Scientific, Waltham, MA, USA). cDNA was transcribed from the RNA with PrimeScript transcriptase reagent kit (Takara, Dalian, China). Subsequently, quantitative real-time polymerase chain reaction (qPCR) analysis was performed using SYBR Premix Ex Taq II (Takara, Dalian, China). The relative expression of mRNA was evaluated using the 2^−ΔΔCt^ method, with β-actin serving as the internal control. The primer sequences utilized for qPCR were listed in Supplementary Table S1.

### 2.6. RNA Interference

Small interfering RNAs (siRNAs) targeting human YTHDF2, MYBL2, UPF1, and the negative control siRNA (siNC) were synthesized by GenePharma (Shanghai, China). TNBC cells were transfected with siRNAs using Lipofectamine RNAiMAX (ThermoFisher, USA) following the manufacturer’s protocol. The specific siRNA sequences were listed in Supplementary Table S2.

### 2.7. Cell Proliferation Assay and Chemosensitivity Assay

For the cell proliferation assay, 2000 transfected cells were seeded in 96-well plates and cultured for different time periods. After that, 10 μL CCK-8 (Cell Counting Kit-8) reagent (Dojindo, Shanghai, China) was added to each well and incubated for 4 h. The optical density (OD) value at 450 nm was measured by a microplate reader. For the chemosensitivity assay, 5000 transfected cells were seeded in 96-well plates. After a 12 h incubation, docetaxel was applied at specified concentrations for 48 h. Subsequently, 10 μL CCK-8 reagent was added to each well, and the cells were incubated for 4 h. The OD value at 450 nm was measured using a microplate reader. Each sample was tested in three replicate wells, and the experiment was performed in triplicate.

### 2.8. RNA Pull-Down Assay

Biotin-labeled sense and antisense GAS5 RNAs were transcribed in vitro using the Biotin RNA Labeling Mix (Roche, Shanghai, China) and T7/SP6 RNA polymerase (Promega Madison, WI, USA) according to the manufacturer’s instructions. The DNA templates were generated by PCR from the pCDH-GAS5 plasmid using primers containing T7 or SP6 promoters. After transcription, the RNA was treated with RNase-free DNase I and purified using an RNeasy Mini Kit (Qiagen, Hilden, Germany). For the pull-down assay, 1 × 10^7^ MDA-MB-231 cells were lysed in IP lysis buffer. Cell lysates were pre-cleared with streptavidin-coated magnetic beads (Thermo Fisher) for 1 h at 4 °C. Pre-cleared lysates were then incubated with 500 ng of biotin-labeled sense or antisense GAS5 RNA for 2 h at 4 °C with rotation, followed by incubation with fresh streptavidin magnetic beads for an additional 1 h. The beads were washed five times with ice-cold wash buffer. One-third of the precipitated material was eluted in 1× SDS loading buffer for Western blot analysis of YTHDF2 (Proteintech, Wuhan, China). The remaining two-thirds were used for RNA extraction with TRIzol, followed by qRT-PCR detection of MYBL2 and GAPDH mRNA. GAPDH served as a negative control for RNA specificity. The fold enrichment of MYBL2 was calculated as (%Input of Sense-GAS5)/(%Input of Antisense-GAS5). Each experiment was performed with three biological replicates.

### 2.9. Transwell Assays

For the migration assay, a total of 1 × 10^5^ cells were cultured in serum-free medium, then cells were seeded in the upper chamber of a Transwell plate. For the invasion assay, the cells were seeded in an extracellular matrix-coated upper chamber. In the bottom chamber, 600 μL of culture medium supplemented with 5% FBS (for the migration assay) or 10% FBS (for the invasion assay) was added. After 24 h, the migrated or invaded cells present on the lower surface of the membrane were stained with 0.1% crystal violet for 15 min, and then their quantities were counted under a microscope.

### 2.10. Chemotaxis Assays

A total of 2 × 10^4^ purified MDSCs isolated from PBMCs of healthy donors were seeded in the upper chamber of a Transwell plate, while different conditioned TNBC cell culture supernatants were added to the lower chamber. After 24 h incubation, the number of migrated cells in the lower chamber was quantified using flow cytometry. The chemotaxis index (migration index) = the number of migrating cells in the test supernatant ÷ the number of migrating cells in the control medium.

### 2.11. Flow Cytometry

Annexin-V-fluorescein isothiocyanate (FITC)/propidium iodide (PI) double staining apoptosis kit (Dojindo Molecular Technologies, Beijing, China) was applied for the cell apoptosis assay. The transfected cells were stained with 5 μL of Annexin V-FITC and 5 μL of PI for 5 min in the dark. Then, cell apoptosis was evaluated using a Flow Cytometer (BD FACSVerse) (BD Biosciences, Milpitas, CA, USA). Single cells were stained with fluorochrome-conjugated antibodies for surface marker analysis for 20–30 min at room temperature in the dark, then analyzed using a Flow Cytometer (BD FACSAria III) (BD Biosciences, Milpitas, CA, USA), and data were analyzed using FlowJo software. The antibodies used in the flow cytometry experiments are listed in Supplementary Table S3.

### 2.12. Western Blot

Cellular proteins were extracted with RIPA lysis buffer (Beyotime Biotechnology, Shanghai, China), and the protein concentration was determined using the BCA kit (Beyotime Biotechnology, Shanghai, China). Following this, the protein samples were separated by 10% SDS-PAGE gels and subsequently transferred onto 0.22 μm PVDF membranes (Millipore, MA, USA). After being incubated with the blocking solution (Beyotime Biotechnology, Shanghai, China) for 1 h, the membranes were incubated with specific primary antibodies overnight at 4 °C. Next, the membranes were incubated with the corresponding secondary antibody for 2 h at room temperature. Protein bands were detected with chemiluminescence reagent (Thermo Fisher). The antibodies utilized in this study were listed in Supplementary Table S3. The original blot can be found in the Supplementary File.

### 2.13. RNA Stability Analysis

Transfected cells were treated with 5 μg/mL of Actinomycin D (Sigma, Darmstadt, Germany) and harvested at 0, 3, 6, 9, or 12 h after Actinomycin D treatment. Then, the total RNA was extracted, and the relative mRNA levels were analyzed by qRT-PCR. The relative mRNA expression at each time point was normalized to the 0 h value. mRNA decay curves were fitted to a one-phase exponential decay model using GraphPad Prism 9.0. Three independent experiments were performed, each with triplicate technical replicates.

### 2.14. RNA Immunoprecipitation (RIP) Assay

RIP assay was performed using the Magna RIP RNA-Binding Protein Immunoprecipitation Kit (Merck Millipore, Darmstadt, Germany) according to the manufacturer’s instructions. Briefly, target cells were lysed and incubated with magnetic beads pre-incubated with YTHDF2 (Proteintech, 24744-1-AP) or control IgG antibody for immunoprecipitation. The enriched RNAs were extracted and analyzed by RT-qPCR.

### 2.15. m6A-RNA Immunoprecipitation (MeRIP)-qPCR Assay

The MeRIP assay was performed using an m6A-methylated RNA Immunoprecipitation Kit (Merck Millipore, Darmstadt, Germany) according to the manufacturer’s instructions. Briefly, RNA was purified and fragmented into ∼100 nt fragments. Then the fragmented RNA was incubated with an anti-m6A primary antibody or an IgG antibody for immunoprecipitation. Next, the m6A-enriched RNAs were analyzed by qPCR assay.

### 2.16. ELISA

Sorted MDSCs (CD11b^+^CD33^+^HLA-DR^−^/low, purity > 95%) were cultured in complete medium for 48 h. Supernatants were collected, and IL-6 concentrations were measured using a human IL-6 ELISA kit (R&D Systems, Shanghai, China). Different conditioned TNBC cells were cultured in 6-well plates, and the supernatant was collected after 48 h. The concentrations of CCL2 in the supernatants of different transfected TNBC cells were detected using an ELISA kit (R&D Systems, USA), according to the manufacturer’s instructions.

### 2.17. Luciferase Reporter Assay

Regarding the MYBL2 and STAT3 binding promoter, cells were co-transfected with luciferase reporter gene plasmids (pGL3-CCL2-WT, pGL3-CCL2-Mut, pGL3-UPF1-WT, or pGL3-UPF1-Mut), OE-MYBL2 plasmid, and internal control pRL-TK plasmid using Lipofectamine 3000 reagent (ThermoFisher, USA). For the detection of binding at the m6A sites, wild-type or mutant plasmids containing m6A sites (pmir-MYBL2-WT or pmir-MYBL2-Mut) were co-transfected with YTHDF2-OE plasmids into TNBC cells using Lipofectamine 3000 reagent (ThermoFisher, USA). After 48 h, the firefly and Renilla luciferase activities were measured using the dual-luciferase reporter assay kit according to the manufacturer’s instructions (Promega, USA); the results were shown as relative luciferase activity.

### 2.18. Chromatin Immunoprecipitation (ChIP) Assays

ChIP assays were performed using the ChIP Assay Kit (Thermo Fisher, USA) according to the manufacturer’s protocol. Briefly, cells from each group were fixed with 1% formaldehyde for 10 min to cross-link the nuclear proteins to DNA. Next, the cells were lysed in SDS lysis buffer followed by sonication. Afterward, the mixture was centrifuged to collect the supernatant, and the anti-MYBL2 antibody (CST, 33056) or an anti-IgG antibody was added for immunoprecipitation, followed by overnight incubation at 4 °C. Subsequently, agarose beads were added to each IP and incubated for 1 h at 4 °C on a rocking platform. After washing, the proteins were eluted from the beads and subjected to heating at 65 °C for 2 h. Finally, the precipitated DNAs were recovered and subjected to qPCR.

### 2.19. Animal Experiments

Four- to six-week-old female BALB/c nude mice were purchased from Beijing Vital River Laboratory Animal Technology Co., Ltd. (Beijing, China) and housed under specific pathogen-free conditions. Prior to inoculation, MDA-MB-231 cells were stably engineered to overexpress or knock down GAS5 via lentiviral transduction, as described in Section 2.2. A total of 5 × 10^6^ MDA-MB-231 cells, with overexpressed or knocked-down GAS5, were suspended in 200 μL of PBS and then subcutaneously injected into the left flank of each mouse. Mice were randomly divided into the following groups (*n* = 5 per group): Vector + PBS, GAS5-OE + PBS, shNC + PBS, shGAS5 + PBS, Vector + DTX, GAS5-OE + DTX, shNC + DTX, shGAS5 + DTX. Beginning 7 days post-inoculation (when palpable tumors were established), mice in the DTX treatment groups received intraperitoneal injections of docetaxel (DTX; Selleck Chemicals, Shanghai, China) at 1 mg/kg body weight dissolved in PBS containing 5% DMSO, administered twice weekly (every 3–4 days) for a total of 4 weeks. Mice in the PBS control groups received equivalent volumes of PBS with 5% DMSO on the same schedule. Tumor size was measured weekly using digital calipers, and tumor volume was calculated according to the formula: tumor volume (mm^3^) = 0.5 × length × width^2^. At the experimental endpoint (4 weeks post-treatment), mice were euthanized, and tumors were excised, weighed, and immediately processed. A portion of each tumor was snap-frozen in liquid nitrogen for qRT-PCR validation of GAS5 expression. The remaining tissue was fixed in 4% paraformaldehyde for paraffin embedding, sectioning, and subsequent immunohistochemical staining (IHC) and flow cytometry analysis of tumor-infiltrating MDSCs. All animal experiments were approved by the Laboratory Animal Welfare and Ethics Committee of the Army Medical University (Approval No. AMUWE2025203).

### 2.20. Immunohistochemistry (IHC)

The harvested xenograft tumor tissues were fixed with 4% polyoxymethylene, then paraffin-embedded and sectioned at a thickness of 4 μm. The tissue sections underwent deparaffinization and rehydration, followed by antigen retrieval and the removal of endogenous peroxidase activity. Subsequently, the sections were incubated with primary antibodies (Ki67, 1:1000; MYBL2, 1:1000; UPF1, 1:1000) overnight at 4 °C. After washing, the sections were incubated with corresponding secondary antibodies. Then, diaminobenzidine was used for immunodetection, and counterstained with hematoxylin. Finally, the sections were observed using a light microscope to capture photos.

### 2.21. Statistical Analysis

Data analysis was performed using GraphPad Prism 9.0. The data are presented as mean ± standard deviation (SD) from three independent experiments. Comparisons between two groups or among multiple groups were performed using Student’s *t*-test or ANOVA, respectively. *p* < 0.05 was considered statistically significant.

## 3. Results

### 3.1. GAS5 Was Expressed at Low Levels in TNBC Cells and Tissues, as Well as in Chemotherapy-Resistant Conditions

To explore the role of GAS5 in the progression of TNBC, we analyzed GAS5 expression in TNBC using the online UALCAN database, and we found lower GAS5 expression in TNBC tissues than in normal tissues (Figure 1A). Subsequently, qRT-PCR confirmed lower expression of GAS5 in TNBC tissues than in adjacent noncancerous tissues of TNBC patients (Figure 1B). In addition, we found that GAS5 level was lower in chemoresistant TNBC tissues than in chemosensitive TNBC tissues (Figure 1C). Compared with normal cells, GAS5 RNA expression in TNBC cells was significantly decreased (Figure 1D). DTX-resistant TNBC cells exhibited even lower levels of GAS5 than TNBC cells (Figure 1D). These results suggest that GAS5 has the potential to become a molecular target for the diagnosis, treatment, and chemotherapy resistance of TNBC.

### 3.2. GAS5 Suppressed the Progression of TNBC Cells and Increased Their Sensitivity to DTX In Vitro

To explore the function of lncRNA GAS5, stable GAS5 overexpression cells were established by transfecting lentivirus carrying GAS5 into TNBC and DTX-resistant TNBC cells, and its overexpression efficiency was validated by qRT-PCR (Figure 2A). CCK-8 and colony formation assays revealed that overexpression of GAS5 inhibited the proliferation of TNBC cells (Figure 2B, Supplementary Figure S2). Transwell assays demonstrated that GAS5 overexpression reduced the migration and invasion abilities in TNBC cells (Figure 2C,D). Conversely, the stable knockdown of GAS5 (Figure 2E) promoted cell growth (Figure 2F), migration, and invasion (Figure 2G,H). Meanwhile, overexpression of GAS5 decreased the IC50 values of DTX in both the parental cells and DTX-resistant cells (Figure 2I,J). Moreover, overexpression of GAS5 weakened the proliferation ability of DTX-resistant TNBC cells (Figure 2K). In addition, as confirmed by flow cytometry, GAS5 overexpression enhanced DTX-induced apoptosis in DTX-resistant TNBC cells (Figure 2L). These results indicate that GAS5 reduces proliferation and increases sensitivity to DTX in TNBC.

### 3.3. GAS5 Repressed MDSCs Infiltration Through YTHDF2/MYBL2/CCL2 Axis in TNBC

To investigate the underlying mechanism by which GAS5 regulates the progression of TNBC, we focused on the tumor microenvironment, and increasing evidence suggests that MDSCs play an important role in the progression of TNBC [3]. Therefore, we further explored the regulatory effect of GAS5 in the interaction between tumor cells and infiltrating MDSCs. As shown in Figure 3A, Transwell migration assays revealed that the migration of MDSCs co-cultured with GAS5-overexpressing TNBC cells was decreased, whereas GAS5 knockdown significantly increased the migration of MDSCs (Figure 3A, Supplementary Figure S3). To explore the mechanism by which GAS5 inhibits the recruitment of MDSCs, we employed the UALCAN database to predict and found that MYBL2 (MYB proto-oncogene like 2), which has been confirmed as an oncogene in breast cancer [15], might be the target gene of GAS5. MYBL2 has been demonstrated to be correlated with immune infiltrates in cancer [16], and MYBL2 was positively correlated with MDSC infiltration in TNBC, which was analyzed by TIMER 2.0 platform (*p* = 1.48 × 10^−3^) (Figure 3B). Therefore, we speculate that GAS5 participates in regulating the infiltration of MDSCs by targeting MYBL2 in TNBC. Using the UALCAN database, we found that MYBL2 expression is higher in TNBC tissues than that in normal tissues (Figure 3C). We also found high expression of MYBL2 in our TNBC samples and negatively correlated with GAS5 expression (Figure 3D,E), suggesting that GAS5 may regulate MYBL2. qRT-PCR and Western blot confirmed that overexpression of GAS5 reduced MYBL2 expression, while knocking down GAS5 increased MYBL2 expression (Figure 3F). However, we did not find the direct binding of GAS5 to MYBL2 through an RIP assay, suggesting that GAS5 might indirectly regulate MYBL2. Our previous research revealed that GAS5 was decreased in breast cancer and regulated by FTO-mediated m6A modification [17]. Therefore, we speculated that GAS5 might regulate MYBL2 by recruiting the m6A reader YTHDF2. As expected, through an RNA pull-down assay, we found that GAS5 simultaneously pulls down both YTHDF2 protein and MYBL2 mRNA, suggesting that all three molecules reside within the same complex (Figure 3G). Using the online database ENCORI and RIP assay, we confirmed the interaction between YTHDF2 and GAS5 (Figure 3H); meanwhile, the SRAMP database analysis revealed that there were potential m6A modification sites in MYBL2. Subsequently, we performed MeRIP-qPCR assays, and the results confirmed that MYBL2 mRNA is an m6A-modified substrate in TNBC cells (Figure 3I). In addition, the luciferase reporter assay showed that YTHDF2 overexpression significantly reduced the luciferase activity of the wild-type reporter (MYBL2-WT), but failed to suppress the mutant reporter (MYBL2-Mut). This demonstrates that YTHDF2-mediated regulation of MYBL2 is dependent on specific m6A modifications (Figure 3J). Given that YTHDF2 regulates the expression of target genes by modulating the stability of target mRNAs, we conducted RNA stability assays. These data demonstrate that both GAS5 and YTHDF2 accelerate MYBL2 mRNA decay, and that YTHDF2 knockdown rescues the destabilizing effect of GAS5 overexpression (Figure 3K). Moreover, both qPCR and Western blot demonstrated that the MYBL2 levels were reduced by GAS5 overexpression; however, when YTHDF2 was simultaneously knocked down, this reduction was rescued (Figure 3L). Conversely, in GAS5 knockdown cells, exogenous overexpression of YTHDF2 still significantly reduced MYBL2 expression, confirming that GAS5 regulates MYBL2 in a YTHDF2-dependent manner (Figure 3M). Furthermore, RIP assay revealed that overexpression of GAS5 promoted the binding of YTHDF2 to MYBL2, while GAS5 depletion significantly attenuated the interaction between YTHDF2 and MYBL2, reinforcing the argument that GAS5 is required for bridging or stabilizing this ternary complex (Figure 3N).

Studies have shown that CCL2, a member of the C-C chemokine family, is crucial in mediating MDSC recruitment and promotes tumor growth, progression, and metastasis [18]. Thus, we speculated whether CCL2 is a downstream effector molecule of MYBL2. Both RT-qPCR and Western blot demonstrated that the expression of CCL2 increased in cells with overexpression of MYBL2, while the expression of CCL2 decreased in cells with knockout of MYBL2 (Figure 3O). ELISA showed that the concentrations of CCL2 were much higher in the supernatants of MYBL2-overexpressing cells, while the CCL2 expression was decreased in the supernatants of MYBL2 knockdown cells (Figure 3O). Moreover, we predicted the potential binding sites of MYBL2 in the promoter region of CCL2 using JASPAR. Then, luciferase reporter assays were performed. As shown in Figure 3P, overexpression of MYBL2 obviously increased the luciferase activity of the wild-type CCL2 promoter (CCL2-WT). Almost completely abolished in the mutant construct (CCL2-Mut), where MYBL2 overexpression resulted in only 1.1-fold induction (Figure 3P). Additionally, ChIP assay showed that endogenous MYBL2 occupies the CCL2 promoter region containing these binding sites in intact cells. (Figure 3Q). To further validate the causal relationship among GAS5, MYBL2, CCL2, and MDSC recruitment, we performed epistasis rescue experiments. As shown in Figure 3R, MYBL2 overexpression significantly reversed the GAS5 overexpression-induced inhibition of MDSC migration, indicating that MYBL2 functions downstream of GAS5 in this pathway (Figure 3R). Additionally, supplementation with recombinant CCL2 (rCCL2) effectively rescued MDSC migration in the conditioned media of GAS5-overexpressing cells (Figure 3S), confirming that CCL2 is the key downstream effector sufficient to overcome GAS5-mediated suppression of MDSC recruitment. Moreover, GAS5-deficient TNBC cells exhibited a diminished ability to recruit MDSCs after treatment with a CCL2 neutralizing antibody(anti-CCL2) (Figure 3T).

To further characterize the MDSC subsets affected by the GAS5/MYBL2/CCL2 axis, we analyzed the migratory response of M-MDSCs and PMN-MDSCs separately. Interestingly, CCL2 preferentially promoted M-MDSC migration compared to PMN-MDSCs, and GAS5 overexpression-induced suppression of MDSC recruitment was more pronounced in the M-MDSC subset. These findings suggest that the GAS5/YTHDF2/MYBL2/CCL2 regulatory axis predominantly modulates M-MDSC trafficking, which may have important implications for immune surveillance in the TNBC microenvironment (Figure 3U). Taken together, these results firmly establish the GAS5/YTHDF2/MYBL2/CCL2 regulatory cascade in controlling MDSC infiltration in TNBC.

### 3.4. MDSCs Promoted the Proliferation of TNBC Cells and Attenuated the Sensitivity of TNBC Cells to DTX, Which Is Related to GAS5

Cancer cells and TME are considered to reciprocally regulate each other and jointly participate in cancer progression. Therefore, we further explored the role of MDSCs in TNBC cell proliferation and chemoresistance. CCK-8 assays revealed that the proliferation ability of the TNBC cells co-cultured with MDSCs isolated from primary TNBC tissues was significantly higher than that of TNBC cells without MDSCs; however, in the presence of tumor MDSCs, overexpression of GAS5 obviously weakened MDSC-induced TNBC cell proliferation (Figure 4A,B). Meanwhile, in the presence of tumor MDSCs, the IC50 value of DTX in TNBC cells strongly increased, while GAS5-overexpressing cells co-cultured with tumor MDSCs showed an inverse effect (Figure 4C,D). Furthermore, flow cytometry analysis indicated that tumor MDSCs led to a significant decrease in DTX-induced TNBC apoptosis, whereas the apoptosis caused by DTX was enhanced in GAS5-overexpressing cells co-cultured with tumor MDSCs compared with control TNBC cells co-cultured with tumor MDSCs (Figure 4E). Overall, these data suggested that the infiltrating MDSCs promoted the proliferation of TNBC cells and TNBC chemoresistance to DTX, and this effect is closely related to GAS5.

### 3.5. MDSC-Derived IL-6 Downregulated GAS5 Through the STAT3/UPF1 Axis

We next questioned whether MDSCs feedback-regulate GAS5 based on our findings described above. To verify this inference, we detected the expression of GAS5 in TNBC cells cultured in the presence or absence of tumor MDSCs from TNBC patients. As shown in Figure 5A, compared with TNBC without tumor MDSCs from TNBC patients co-cultured, the expression of GAS5 in TNBC co-cultured with MDSCs was significantly decreased (Figure 5A). To understand the mediator contributing to GAS5 downregulation in TNBC co-cultured with tumor MDSCs, we focused on the cytokine IL-6 secreted by MDSCs that can promote the progression of TNBC, which has been reported in previous studies [19]. In our study, we first determined whether MDSCs are a direct source of IL-6; we measured IL-6 secretion by sorted MDSCs. As shown in Figure 5B, both healthy donor-derived and tumor-derived MDSCs secreted detectable levels of IL-6 as quantified by ELISA. Notably, tumor-derived MDSCs secreted significantly higher levels of IL-6 compared to healthy donor-derived MDSCs, consistent with the enhanced inflammatory phenotype of tumor-infiltrating MDSCs. TNBC cells alone also produced IL-6, albeit at lower levels than tumor-derived MDSCs (Figure 5B). qRT-PCR analysis further confirmed IL-6 mRNA expression in MDSCs and TNBC cells, with tumor-derived MDSCs exhibiting the highest expression (Figure 5C). To confirm that MDSC-derived IL-6 is responsible for GAS5 downregulation, we treated TNBC cells with conditioned medium from tumor-derived MDSCs (CM-MDSCs). CM-MDSCs significantly downregulated GAS5 expression, and this effect was largely reversed by pre-incubation with an IL-6 neutralizing antibody, but not with an IgG control. Recombinant IL-6 (rIL-6) phenocopied the effect of CM-MDSCs, confirming IL-6 as the key MDSC-derived factor mediating GAS5 downregulation (Figure 5D). Collectively, these results establish that MDSCs are a direct source of IL-6, and that MDSC-derived IL-6 mediates the downregulation of GAS5 in TNBC cells. Moreover, the CCK8 assay showed that the cell proliferation and DTX resistance induced by MDSCs were weakened by the administration of an IL-6 neutralizing antibody (Figure 5E,F). For in-depth mechanistic insights, we focused on nonsense-mediated mRNA decay (NMD), which was regarded as a crucial RNA surveillance mechanism [20]. By utilizing the TCGA database, we found that up-frameshift suppressor 1 (UPF1), which plays an important role in NMD, was highly expressed in TNBC (Figure 5G). Similarly, the expression of UPF1 in the tissues of TNBC patients was significantly higher than that in the adjacent noncancerous tissues (Figure 5H). Additionally, correlation analysis revealed that the expression of UPF1 and GAS5 were negatively correlated in TNBC (Figure 5I). We examined the expression of GAS5 after overexpressing or knocking down UPF1. The results showed that knocking down UPF1 significantly promoted the expression of GAS5, while overexpressing UPF1 reduced the expression of GAS5 in TNBC (Figure 5J). Then, the RIP assays and RNA stability analysis were performed. The results showed that UPF1 physically associates with GAS5 (Figure 5K). Also, UPF1 overexpression accelerates GAS5 decay, whereas UPF1 knockdown stabilizes GAS5 and prolongs its half-life (Figure 5L). All of these further validate the role of UPF1 in NMD-mediated regulation of GAS5. Interestingly, we observed that the expression of UPF1 increased in TNBC after co-culture with MDSCs, while the expression of UPF1 in TNBC co-cultured with MDSCs decreased with an IL-6 neutralizing antibody (Figure 5M). The IL-6/STAT3 pathway plays an important role in promoting TNBC progression [21], and STAT3 functions as an important transcriptional factor regulating the expression of many target genes by binding to the promoters of target genes [22]. We wondered whether the IL-6/STAT3 pathway transcriptionally regulates UPF1; our investigation revealed that an IL-6 neutralizing antibody inhibited the ability of IL-6 to enhance the levels of p-STAT3 and UPF1. Additionally, treatment with a STAT3 inhibitor (Stattic) abolished the effect of IL6 secreted by MDSCs on UPF1 in TNBC co-cultured with MDSCs (Figure 5N). Subsequently, we predicted the potential binding sites of STAT3 on the UPF1 promoter using JASPAR. Then, the luciferase reporters containing the STAT3 binding sites (wild-type and mutant) in the UPF1 promoter were constructed. In the UPF1 promoter luciferase assay, wild-type UPF1 promoter activity in MDA-MB-231 cells co-cultured with MDSCs was increased compared to the control group without MDSC co-culture, and this effect was weakened by the IL-6 neutralizing antibody. However, no difference was observed in the luciferase activity of the mutant reporter (Figure 5O). Consistently, ChIP analysis revealed that IL-6 secreted by MDSCs significantly enhanced the binding of STAT3 to the UPF1 promoter (Figure 5P). Furthermore, deprivation of UPF1 inhibited the MDSC-mediated decrease in GAS5 (Figure 5Q). In STAT3-depleted cells co-cultured with MDSCs, MDSCs lost the ability to induce UPF1 expression in cells (Figure 5R), thereby leading to an increase in the expression of GAS5 (Figure 5S). These collective observations indicated that MDSCs feedback-downregulate GAS5 through the IL-6/STAT3/UPF1 axis.

### 3.6. Overexpression of GAS5 Suppressed the Growth, DTX Resistance, and MDSCs Recruitment in TNBC In Vivo

To further elucidate the functional significance of GAS5 in TNBC progression, the cells overexpressing or knocking down GAS5 were subcutaneously injected into the flanks of BALB/c nude mice to generate tumor xenografts, followed by administration with PBS or DTX. As shown in Figure 6, overexpression of GAS5 resulted in decreased tumor weight and volume compared with the control group (Figure 6A–C). Meanwhile, in mice, simultaneous GAS5 overexpression together with DTX treatment exhibited significant inhibition of tumor growth (Figure 6A–C), suggesting that overexpression of GAS5 enhanced DTX sensitivity in vivo. Conversely, GAS5 knockdown led to an increase in tumor growth compared with control cells, and weakened the anti-tumor effect of DTX in vivo (Figure 6D–F). The qRT-PCR analysis of the isolated tumor samples revealed that the intratumoral levels of MYBL2 and UPF1 were significantly reduced in the OE-GAS5 group, and a further decline was observed in the overexpressed GAS5 together with DTX treatment group (Figure 6G,H). ELISA analysis revealed that the level of chemokine CCL2 in tumor tissue lysates was significantly lower in the OE-GAS5 group than that in the control group (Figure 6I). IHC analysis of the tumor tissues revealed that the intratumoral expression levels of Ki67, MYBL2, and UPF1 were notably decreased in the OE-GAS5 group, with a further reduction observed in the OE-GAS5 together with DTX treatment group (Figure 6J). Moreover, flow cytometry analysis confirmed a significant decrease in infiltrated MDSCs (CD11b^+^Gr1^+^) in tumor tissues with high GAS5 expression. Meanwhile, DTX treatment further reduced the infiltration of MDSCs in tumor tissues with GAS5 overexpression (Figure 6K). Furthermore, knockdown of GAS5 produced the opposite effects (Figure 6G–K). These results indicate the role of GAS5 in modulating tumor growth, chemotherapy sensitivity, and MDSC infiltration in TNBC.

## 4. Discussion

Compared with other subtypes of breast cancer, TNBC is characterized by high relapse, metastases, and chemoresistance, all of which contribute to the high mortality and poor prognosis of TNBC patients. Revealing novel targets is of great significance for understanding the underlying mechanisms and developing effective treatment strategies, ultimately improving the prognosis of TNBC patients.

LncRNA GAS5 plays a role as a tumor suppressor in various tumors, and its down-regulation is closely related to tumor progression, chemotherapy resistance, and poor prognosis. Emerging evidence has demonstrated that GAS5 competitively binds to multiple oncogenic miRNAs through complementary sequences, thereby relieving the inhibition of these miRNAs on the downstream tumor suppressor target genes in various tumors [23,24]. Additionally, GAS5 has been found to regulate tumor progression through epigenetic mechanisms by recruiting target proteins. For example, overexpression of GAS5 inhibits cell proliferation partially through interacting with and suppressing androgen receptor activity in prostate cancer [25]. In ovarian cancer (OC), GAS5 significantly increased the sensitivity of OC cells to cisplatin and suppressed OC progression by recruiting the transcription factor E2F4 to the PARP1 promoter and affecting the activity of the MAPK pathway [26]. Previous reports have revealed that low levels of GAS5 are closely related to the malignant progression of tumors, poor prognosis, and chemotherapy tolerance [10]. Consistent with previous studies, we found that GAS5 was expressed at a low level in TNBC tissues and cells; moreover, its expression is even lower in chemoresistant TNBC tissues and cells, which showed a close correlation between decreased GAS5 and TNBC progression. Our functional experiments indicate that GAS5 suppressed the progression of TNBC and increased their sensitivity to DTX in vitro. We acknowledge that the current study focused exclusively on docetaxel (DTX) as the chemotherapeutic agent, and therefore our conclusions regarding GAS5-mediated chemosensitivity cannot be directly extrapolated to other routinely used agents in TNBC. Clinically, TNBC is treated with diverse regimens, including platinum-based drugs (e.g., carboplatin, cisplatin), anthracyclines (e.g., doxorubicin), and cyclophosphamide, either as monotherapies or in combination. Whether GAS5 modulates cellular sensitivity to these alternative agents remains unaddressed. Given that GAS5 functions, at least in part, through regulation of the tumor microenvironment and MDSC infiltration, it is plausible that the GAS5-mediated feedback loop could influence sensitivity to other chemotherapeutics that are similarly affected by immunosuppressive microenvironments or DNA damage repair pathways. However, this hypothesis requires direct experimental validation. Future studies are encouraged to systematically evaluate whether GAS5 overexpression or knockdown alters the IC_50_ values of platinum compounds, anthracyclines, and alkylating agents in TNBC cell lines, and to assess whether the YTHDF2/MYBL2/CCL2 axis and the MDSC-mediated feedback loop operate uniformly across different drug classes.

Growing evidence indicates that TME plays a crucial role in determining the fate of cancer cells. Therefore, targeting TME in combination with anti-tumor therapy has become a research hotspot. The increased numbers of MDSCs within the TME play an important role in tumor progression [27]. Previous studies found that the content of MDSCs in the TNBC subtypes is higher than that in the non-TNBC subtypes [28]. The MDSCs recruited to the TME promoted TNBC tumor growth, progression, metastasis, and chemotherapy resistance [29,30]. In this study, we showed that overexpression of GAS5 contributes to inhibiting the recruitment of MDSCs to the TME. Mechanistically, our studies demonstrated that GAS5 represses MDSC recruitment through the YTHDF2/MYBL2/CCL2 axis. CCL2, produced by both tumor cells and non-tumor cells, regulates MDSC recruitment and function, which promotes tumor growth, progression, and metastasis [31]. In this study, we observed that MYBL2 transcriptionally activated CCL2 and enhanced CCL2 expression, which further promoted the recruitment of MDSCs. MYBL2 is an important transcription factor that plays a central role as an oncogene in the occurrence, development, treatment, and prognosis of TNBC [15,32]. MYBL2 is highly expressed in BC tissues and cells, and this high level is significantly associated with shorter overall survival, poorer survival, and higher metastasis risk [15,16]. In addition, MYBL2 has been demonstrated to be correlated with immune infiltrates in tumors, and MYBL2 was positively correlated with MDSCS infiltration in TNBC [33]. In our present research, we found that the expression of GAS5 was negatively correlated with MYBL2 in TNBC samples, and overexpression of GAS5 reduced MYBL2 expression, thereby reducing the recruitment of MDSCs. Subsequent investigations explored the mechanism by which GAS5 regulates the recruitment of MDSCs mediated by MYBL2. Interestingly, we found that GAS5 indirectly leads to MYBL2 instability and reduces its expression by promoting the binding of YTHDF2 to MYBL2. As an m6A reader, the core function of YTHDF2 is to recognize and bind to target mRNAs with m6A modification, and it exerts its biological effects by reducing their stability and promoting their degradation [34]. In this study, we revealed that YTHDF2-mediated regulation of MYBL2 is dependent on specific m6A modifications, and MYBL2 stability and expression were reduced by GAS5 overexpression in a YTHDF2-dependent manner, suggesting that GAS5 acts as a molecular scaffold that recruits YTHDF2 to specific m6A sites on MYBL2 mRNA, thereby accelerating its degradation.

MDSCs, as the major component of the TME, are a heterogeneous, immature plastic population of myeloid cells that are divided into two main classifications of MDSCs, granulocytic/polymorphonuclear (PMN-MDSCs) and monocytic (M-MDSCs), with distinct surface markers [14]. In our study, to further characterize the MDSC subsets affected by the GAS5/MYBL2/CCL2 axis, we analyzed the migratory response of M-MDSCs and PMN-MDSCs separately. Both subsets exhibited immunosuppressive activity in T-cell proliferation assays. Interestingly, CCL2 preferentially promoted M-MDSC migration compared to PMN-MDSCs, and GAS5 overexpression-induced suppression of MDSC recruitment was more pronounced in the M-MDSC subset. These findings suggest that the GAS5/YTHDF2/MYBL2/CCL2 regulatory axis predominantly modulates M-MDSC trafficking, which may have important implications for immune surveillance in the TNBC microenvironment. We acknowledge certain limitations in our study. First, while we characterized MDSC subsets using HLA-DR, CD14, and CD15 markers, and confirmed their immunosuppressive function via T-cell proliferation assays, our primary phenotypic definition of MDSCs (CD11b^+^CD33^+^HLA-DR^−^/low) may not capture the full heterogeneity of MDSC populations. Second, the MDSCs used in chemotaxis assays were derived from healthy donor PBMCs, whereas those used in co-culture experiments were derived from primary TNBC tissues. Although both populations were functionally validated as immunosuppressive, and both exhibited similar trends in our assays, we acknowledge that tumor-derived MDSCs may possess distinct functional properties compared to their peripheral counterparts. Future studies using patient-matched peripheral and tumor-derived MDSCs would provide a more comprehensive understanding of MDSC biology in TNBC.

Recent studies implicate the complex interplay between infiltrating immune cells and cancer cells in tumor progression [35]. This interaction involves direct contact and cytokine release, thereby influencing tumor progression and suggesting innovative treatment strategies [6,19]. Here, we observed that the low expression of GAS5 in TNBC cells led to an increase in the secretion of chemokine CCL2, which in turn led to the accumulation of MDSCs in the tumor microenvironment, thereby promoting tumor progression. This effect highlights the crucial role of GAS5-low-expressing TNBC in recruiting MDSC cells to the tumor microenvironment, promoting the progression of TNBC and facilitating a deeper study of the mechanism of the anti-tumor effect of GAS5. On the other hand, previous literature reported that MDSCs directly affect characteristics of tumor cells through the release of cytokines, which affects the therapeutic outcome and provides a basis for innovative treatment strategies [6,19]. Consistent with this, we observed that MDSC-secreted IL-6 was essential for GAS5 downregulation and promoted tumor growth and chemoresistance in TNBC, which established a feedback loop between MDSCs and TNBC cells. Mechanistically, our studies indicated that MDSCs feedback-downregulate GAS5 through the IL-6/STAT3/UPF1 axis. STAT3 is a cytoplasmic transcription factor that plays a critical role in cell proliferation, apoptosis, and differentiation [36]. Diverse human malignancies exhibit an activated IL-6/STAT3 signaling pathway, which is related to the progression and metastasis of tumors [37]. Many human malignant tumors exhibit an activated IL-6/STAT3 pathway, which is closely related to the progression of the tumors. In our study, a specific binding site of STAT3 was identified in the UPF1 promoter region, indicating that the transcription of UPF1 is directly regulated by the activation of the IL-6/STAT3 pathway. UPF1, an essential NMD factor, is an RNA-binding protein that participates in multiple pathways involving RNA degradation, which involves not only protein-coding genes but also long non-coding RNAs [38]. It has been reported that knocking out UPF1 remarkably stabilizes and upregulates GAS5 [39,40]. In this study, we found that UPF1 binds to GAS5 and causes the degradation of GAS5, which validates the role of UPF1 in NMD-mediated regulation of GAS5. Also, the expression of UPF1 increased in TNBC after co-culture with MDSCs, while the expression of UPF1 in TNBC co-cultured with MDSCs decreased with an IL-6 neutralizing antibody. Furthermore, our investigation revealed that anti-IL-6 inhibited the ability of IL6/STAT3 pathway to enhance the level of UPF1; additionally, treatment with a STAT3 inhibitor (Stattic) abolished the effect of IL-6 secreted by MDSCs on UPF1 in TNBC co-cultured with MDSCs. Moreover, deprivation of UPF1 inhibited the MDSC-mediated decrease in GAS5.

We acknowledge several limitations regarding our in vivo experiments. The xenograft model using BALB/c nude mice, while appropriate for studying human TNBC cell growth and MDSC recruitment, lacks adaptive immunity (T cells) and may not fully recapitulate the complex tumor-immune microenvironment of TNBC patients. Although nude mice retain functional innate immune components, including MDSCs, macrophages, and granulocytes, the absence of T cells limits our ability to assess the impact of GAS5 on adaptive immune responses or the broader immune microenvironment, including potential effects on CD8^+^ T cell infiltration, Treg populations, or antigen-presenting cells. Our findings primarily demonstrate that GAS5 regulates MDSC accumulation in vivo, as supported by flow cytometry analysis of CD11b^+^Gr1^+^ cells in tumor tissues. However, we cannot exclude the possibility that GAS5 may also influence other immune compartments through additional mechanisms. Future studies utilizing syngeneic immunocompetent TNBC models (e.g., 4T1 in BALB/c mice or E0771 in C57BL/6 mice) would be valuable to fully elucidate the role of GAS5 in shaping the broader tumor immune microenvironment and to validate our findings in an immune-competent setting. Furthermore, such models would allow for assessment of whether GAS5-mediated modulation of MDSC recruitment translates into enhanced anti-tumor T cell immunity and improved efficacy of immunotherapies. Despite these limitations, our in vivo data, in combination with our in vitro co-culture experiments using primary human MDSCs, provide compelling evidence for the GAS5/YTHDF2/MYBL2/CCL2 axis in regulating MDSC trafficking and tumor progression in TNBC.

## 5. Conclusions

In summary, we observed a significant downregulation of GAS5 in TNBC tissues, correlated with chemoresistance in TNBC patients. GAS5 overexpression inhibited the progression of TNBC and increased sensitivity to chemotherapy. Mechanistically, GAS5 repressed MDSC infiltration through the YTHDF2/MYBL2/CCL2 axis in TNBC. Reciprocally, infiltrated MDSCs promoted the proliferation of TNBC cells and attenuated the sensitivity of TNBC cells to DTX through the IL-6/STAT3/UPF1 signaling. These findings depicted a feedback loop between TNBC cells and MDSCs; we hypothesize that the sequence is initiated by tumor-intrinsic GAS5 downregulation. The loss of GAS5 derepresses the YTHDF2/MYBL2/CCL2 axis, creating a chemotactic signal for initial MDSC recruitment. Once infiltrated, MDSCs secrete IL-6 to activate the STAT3/UPF1 pathway, which further degrades GAS5 and locks the system into a self-amplifying vicious cycle. Overall, our study illustrated the significance of the GAS5-mediated mutual regulatory pathway between tumor cells and MDSCs in TNBC and provides new avenues for the development of targeted therapies in TNBC.

## Figures and Tables

**Figure 1:**
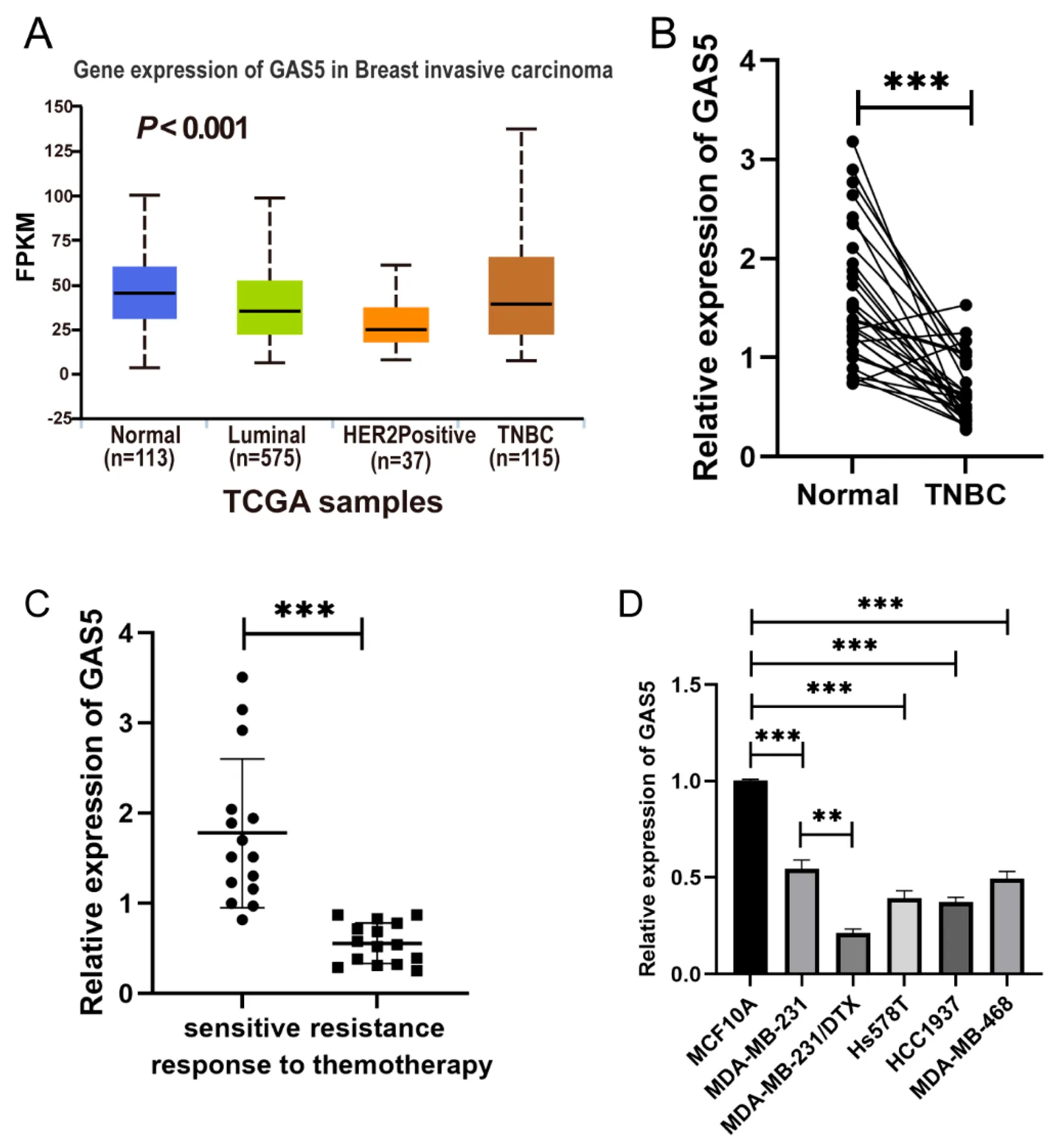
Low levels of GAS5 correlated with chemoresistance in TNBC. (**A**) GAS5 expression in TNBC was analyzed according to the UALCAN database. (**B**) The expression of GAS5 in 30 TNBC tissues and paired adjacent non-tumor normal tissue was determined by RT-qPCR. (**C**) The levels of GAS5 in chemosensitive (*n* = 15) and chemoresistant (*n* = 15) TNBC tissues. (**D**) The GAS5 RNA expression was detected by qRT-PCR in TNBC cells, DTX-resistant TNBC cells, and normal mammary cells (MCF-10A). ** *p* < 0.01, *** *p* < 0.001.

**Figure 2:**
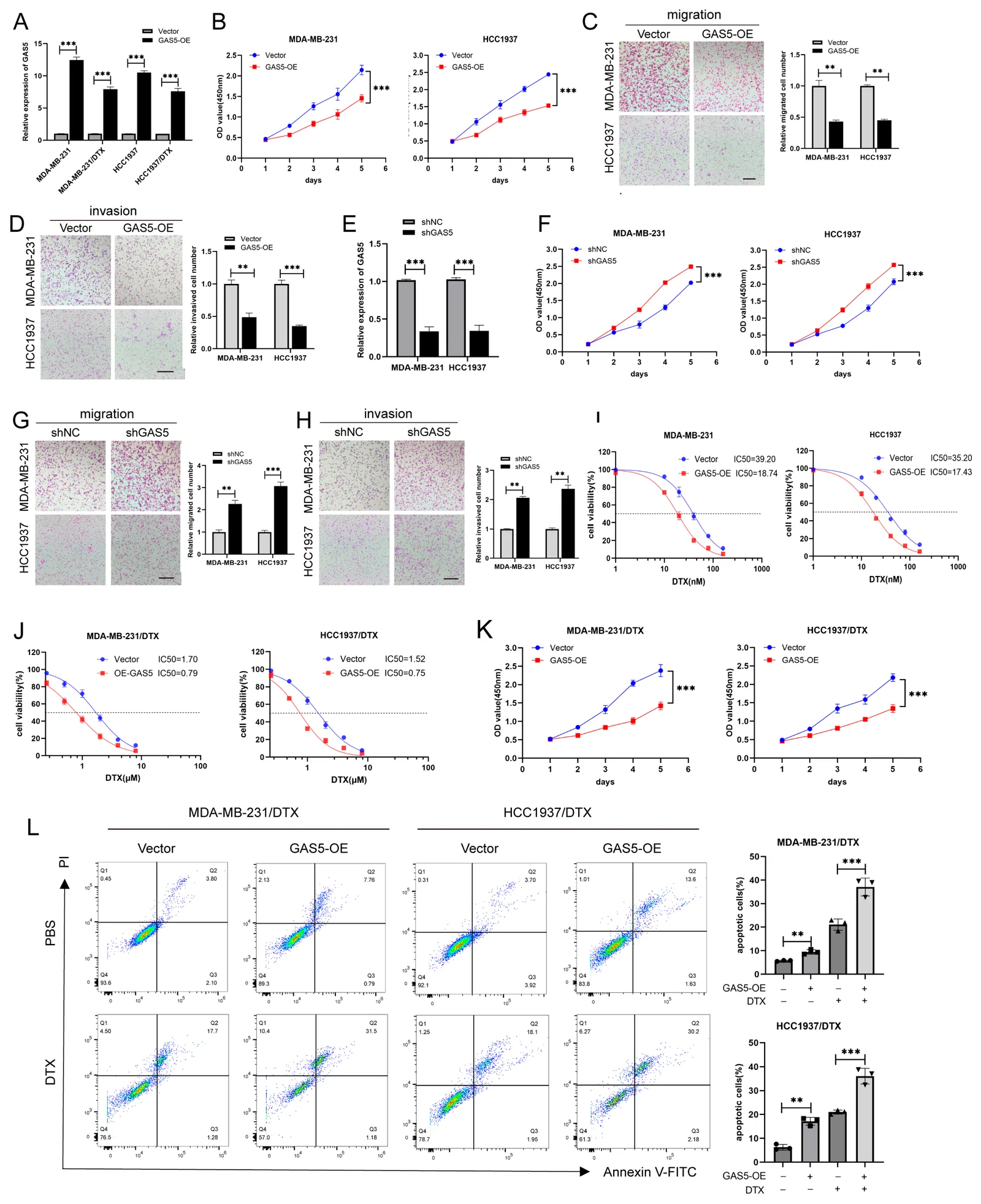
GAS5 overexpression inhibited the progression of TNBC cells and increased their sensitivity to DTX. (**A**) Stable GAS5 overexpression cells were established by transfecting lentivirus carrying GAS5 into TNBC and DTX-resistant TNBC cells, and then an RT-qPCR assay was performed to determine the RNA level of GAS5 in cells with stable overexpression of GAS5. (**B**) CCK-8 assays of GAS5-overexpressing cells were performed to evaluate the effect of GAS5 on cell proliferative ability. (**C**,**D**) The migration (**C**) and invasion (**D**) ability of GAS5-overexpressing cells were assessed by the Transwell assay. Scale bar = 200 μm. (**E**) MDA-MB-231 and HCC1937 cells were transduced with lentiviral shGAS5 or shNC to establish stable GAS5 knockdown cells. Then, RT-qPCR detection of the RNA expression of GAS5 in stable GAS5 knockdown TNBC cells. (**F**) The proliferative ability of GAS5 knockdown cells was detected by CCK-8 assays. (**G**,**H**) The migration (**G**) and invasion (**H**) ability of GAS5 knockdown cells were detected by Transwell assays. Scale bar = 200 μm. (**I**,**J**) The IC50 value of DTX and cell viability were calculated by the CCK-8 assay. (**K**) The proliferative ability of GAS5-overexpressing MDA-MB-231/DTX and HCC1937/DTX cells was assessed by the CCK-8 assay. (**L**) The apoptotic rate of MDA-MB-231/DTX and HCC1937/DTX cells with overexpressed GAS5 with or without DTX was evaluated by flow cytometry. **, *p* < 0.01; ***, *p* < 0.001.

**Figure 3:**
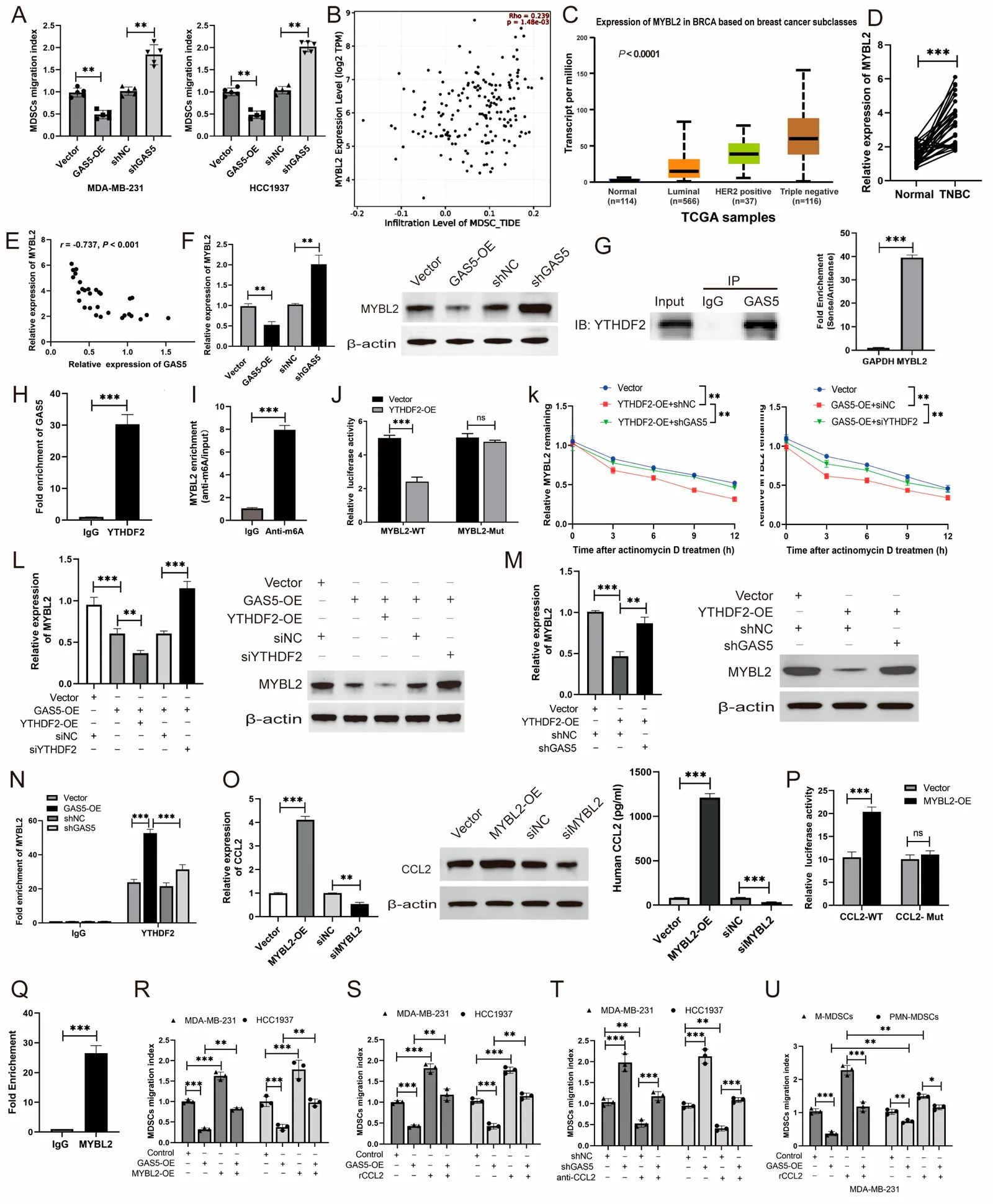
GAS5 repressed MDSC infiltration through the YTHDF2/MYBL2/CCL2 axis. (**A**) PBMCs were isolated from healthy donors, and the MDSCs (CD11b^+^CD33^+^) were sorted by flow cytometry (*n* = 5). The migration index of MDSCs in Transwell assays using conditioned media from GAS5-overexpressing or GAS5-knockdown TNBC cells. (**B**) Correlation between MYBL2 and MDSC infiltration in TNBC samples analyzed by TIMER 2.0. (**C**) MYBL2 expression in TNBC samples analyzed by the UALCAN database. (**D**) The expression of MYBL2 in 30 TNBC tissues and paired adjacent non-tumor normal tissue. (**E**) Correlation between MYBL2 and GAS5 in 30 TNBC tissues analyzed by Pearson correlation analysis. (**F**) qRT-PCR and Western blot were used to measure MYBL2 levels in MDA-MB-231 cells that overexpress or have GAS5 knocked down. (**G**) RNA pull-down assay demonstrating that biotin-labeled sense GAS5 simultaneously captures YTHDF2 protein and MYBL2 mRNA. Left: Western blot showing YTHDF2 enrichment in the sense-GAS5 pull-down pellet compared to the antisense control; Input represents 10% of the total cell lysate. Right: qRT-PCR quantification of MYBL2 and GAPDH enrichment in sense-versus antisense-GAS5 pull-down complexes. The fold enrichment of MYBL2 was calculated as (% Input of Sense-GAS5)/(% Input of Antisense-GAS5). (**H**) The enrichment of GAS5 by IgG and YTHDF2 was detected by RIP assay. (**I**) MYBL2 m6A modification level in MDA-MB-231 cells was analyzed by MeRIP-qPCR assay. (**J**) MDA-MB-231 cells were co-transfected with the indicated dual-luciferase reporter and YTHDF2-OE plasmid or vector, and the relative luciferase activity was measured. The firefly luciferase activity was normalized to Renilla luciferase activity. (**K**) MDA-MB-231 cells stably overexpressing or knocked down GAS5 were transiently transfected with the YTHDF2 overexpression plasmid (YTHDF2-OE), vector, siYTHDF2, or siNC, then treated with actinomycin D (5 μg/mL) to block de novo transcription for the indicated duration, followed by a qRT-PCR assay to determine the stability of MYBL2. (**L**,**M**) qRT-PCR (**L**) and Western blot (**M**) to evaluate the expression of MYBL2, confirming that GAS5 regulates MYBL2 in a YTHDF2-dependent manner. (**N**) The effect of GAS5 on the combination of YTHDF2 with MYBL2 was examined by RIP assay. (**O**) MYBL2 promoted the expression of CCL2, which was detected by qRT-PCR, Western blot, and ELISA. (**P**,**Q**) MYBL2 binds to the promoter region of CCL2, which was detected by luciferase reporter assays (**P**) and ChIP assay (**Q**). (**R**) Chemotaxis assay showing MDSC migration index in response to conditioned media from MDA-MB-231 and HCC1937cells with Control, GAS5-OE, or GAS5-OE + MYBL2-OE. (**S**) Migration index of MDSCs in Transwell assays using conditioned media from GAS5-OE or Control TNBC cells with or without the recombinant CCL2 (rCCL2) (50 ng/mL). (**T**) Migration index of MDSCs in Transwell assays using conditioned media from shGAS5 or shNC TNBC cells with or without the anti-CCL2 antibody. (**U**) Migration index of M-MDSCs or PMN-MDSCs in Transwell assays using conditioned media from GAS5-OE or Control MDA-MB-231 cells with or without rCCL2 (50 ng/mL). The data are presented as the mean ± SD (*n* = 3 independent experiments). n.s., not significant; * *p* < 0.05; ** *p* < 0.01; *** *p* < 0.001.

**Figure 4:**
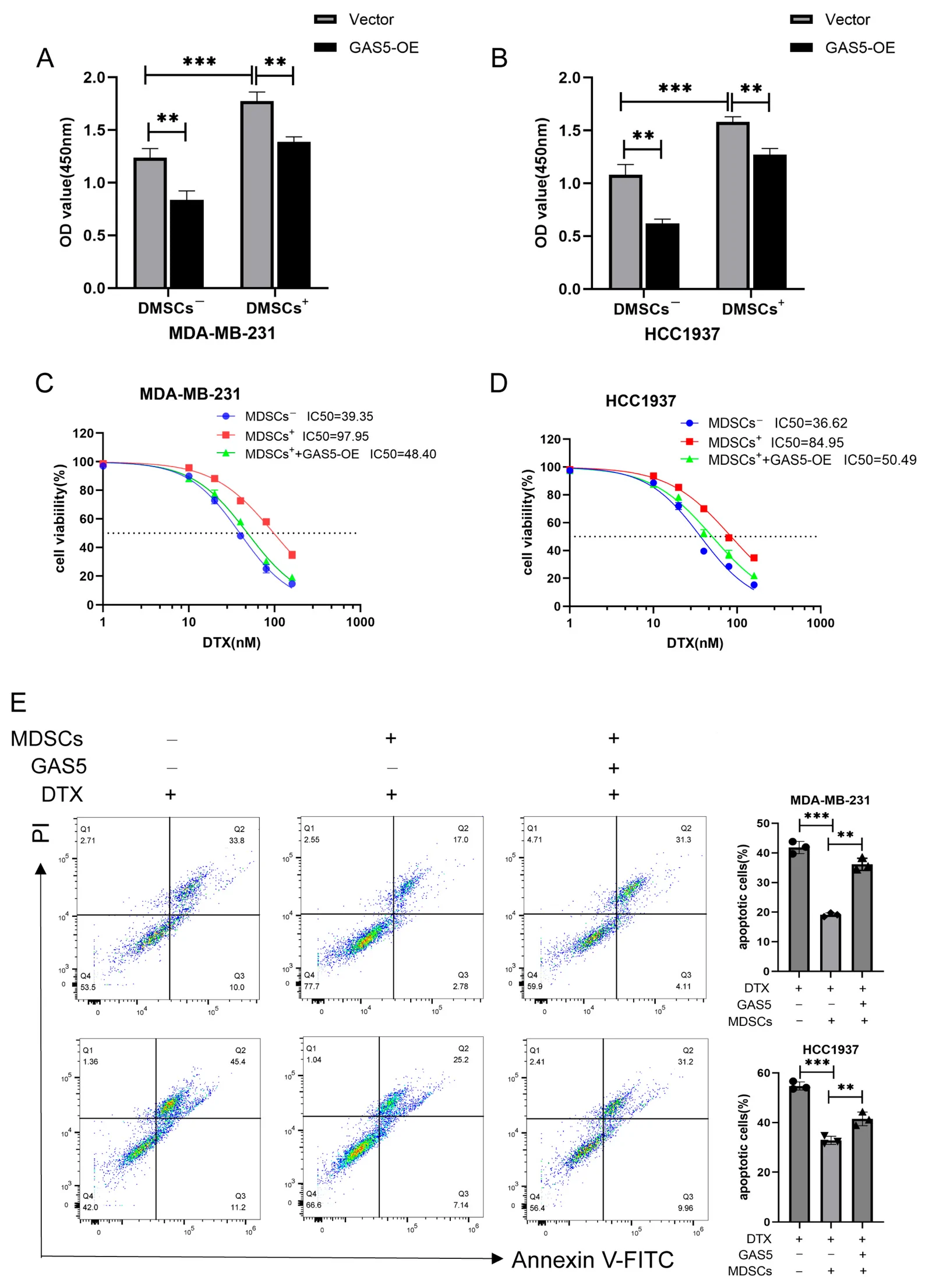
MDSCs promoted the proliferation of TNBC cells and TNBC chemoresistance to DTX. (**A**,**B**) CCK-8 assay on MDA-MB-231 and HCC1937 cells with vector or GAS5-OE after co-culture with or without MDSCs from TNBC tissues (*n* = 3 independent patients) in Transwell for 4 days. (**C**–**E**) Cells overexpressing GAS5 were co-cultured with or without MDSCs from TNBC tissues (*n* = 3 independent patients) for 48 h, and then the cells were treated with DTX for another 48 h. The IC50 value of DTX and cell viability were calculated by CCK-8 assay (**C**,**D**); flow cytometry was performed to examine the effect of MDSCs on DTX-induced apoptosis (**E**). **, *p* < 0.01; ***, *p* < 0.001.

**Figure 5:**
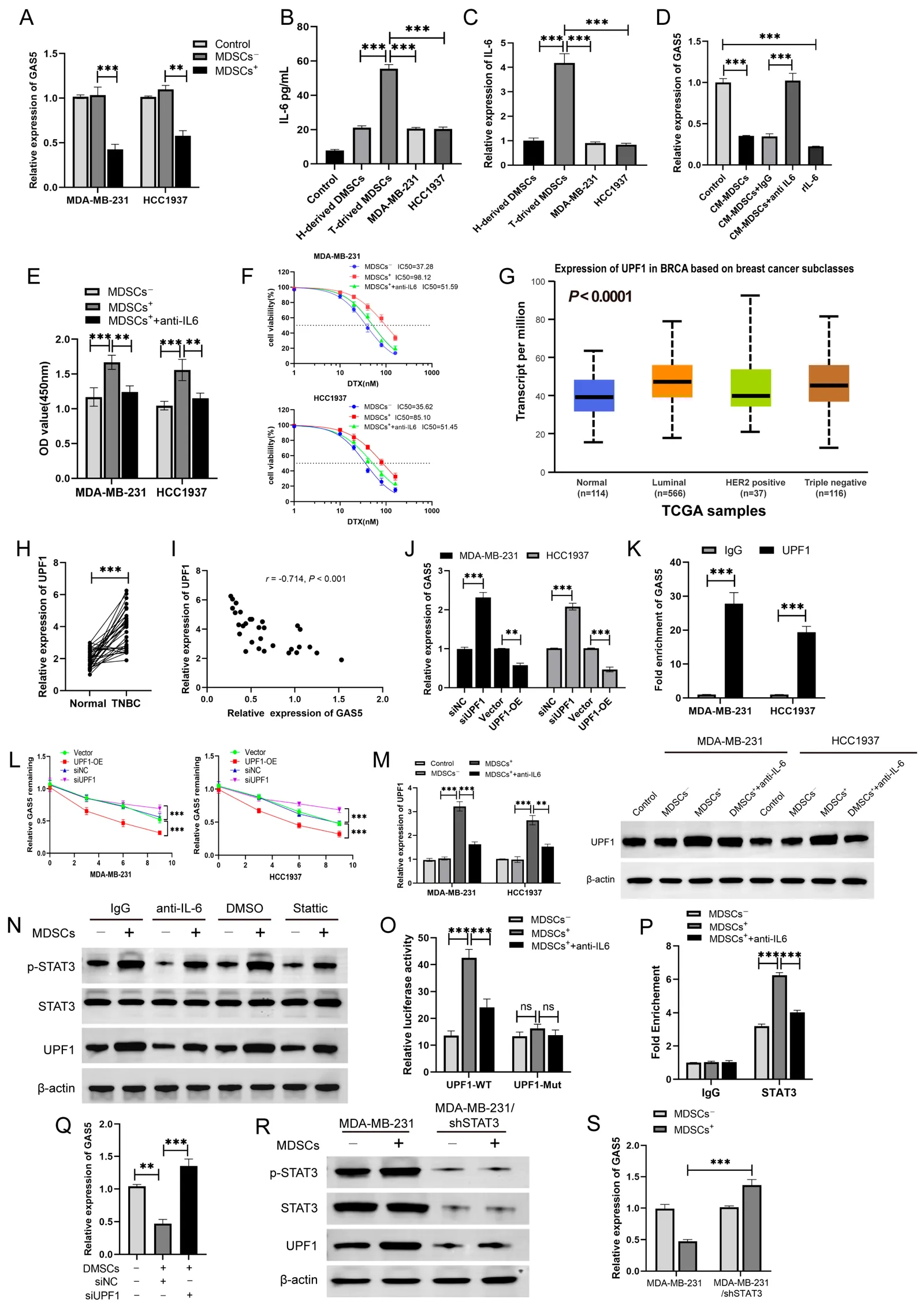
MDSC-derived IL-6 downregulated GAS5 through STAT3/UPF1 axis. (**A**) Cells co-cultured with or without MDSCs from primary TNBC tissues (*n* = 6 independent patients) for 48 h; the expression of GAS5 was measured by qRT-PCR. (**B**) Healthy donor-derived MDSC, tumor-derived MDSCs were sorted and cultured in complete medium for 48 h; the supernatants were collected and used to detect the level of IL-6 using a human IL-6 ELISA kit. Supernatants from MDA-MB-231 and HCC1937 cultured under identical conditions were included as a comparison. Culture medium alone served as a negative control. (**C**) IL-6 mRNA in healthy donor-derived and tumor-derived MDSCs, MDA-MB-231, and HCC1937 cells was measured by qRT-PCR. (**D**) Tumor-derived MDSCs were cultured alone for 48 h, and conditioned medium (CM-MDSCs) was collected, then MDA-MB-231 cells were treated with control medium, CM-MDSCs, CM-MDSCs pre-incubated with IL-6 neutralizing antibody (100 ng/mL), CM-MDSCs pre-incubated with IgG control, and recombinant human IL-6 (rIL-6) (10 ng/mL; positive control) for 24 h, followed by qRT-PCR to dectet the GAS5 expression. (**E**,**F**) TNBC cells co-cultured with or without tumor-derived MDSCs. The addition of an IL-6 neutralizing antibody was performed in the presence of tumor-derived MDSCs for 24 h, and then the cells were treated with different concentrations of DTX for 48 h. Then, cell proliferation (**E**) and the IC50 value of DTX (**F**) were examined by the CCK-8 assay. (**G**) UPF1 expression in TNBC was analyzed according to the TCGA database. (**H**) The expression of UPF1 in 30 TNBC tissues and paired adjacent non-tumor normal tissue was detected by qRT-PCR. (**I**) The correlation between UPF1 and GAS5 expression in breast cancer tissues was analyzed by Pearson’s correlation coefficient. (**J**) Cells were transiently transfected with siUPF1 (50 nM) or a UPF1 overexpression plasmid using Lipofectamine 3000. After 48 h, qRT-PCR was used to detect the level of GAS5 in cells with overexpressed or knocked-down UPF1. (**K**) Binding of UPF1 to GAS5 in MDA-MB-231 and HCC1937 cells was verified by RIP assay. (**L**) Cells with UPF1 overexpression or knockdown were treated with actinomycin D (5 μg/mL) to block de novo transcription for the indicated duration, and GAS5 levels will be quantified by qRT-PCR. (**M**) MDA-MB-231 cells were co-cultured with or without MDSCs from primary TNBC tissues, with an IL-6 neutralizing antibody (100 ng/mL) in MDSC cells; UPF1 levels were measured by qRT-PCR and Western blot. (**N**) Tumor MDSC-derived IL-6 increased UPF1 expression through STAT3. The proteins of interest were detected by Western blot. (**O**) MDA-MB-231 cells were co-transfected with the indicated dual-luciferase reporter, co-cultured with or without MDSCs from primary TNBC tissues, and the relative luciferase activity was measured. The firefly luciferase activity was normalized to Renilla luciferase activity. (**P**) MDSC-derived IL-6 enhanced the binding of STAT3 to the UPF1 promoter was detected by ChIP analysis. (**Q**) MDA-MB-231 cells were transfected with siUPF1 (50 nM) for 24 h prior to co-culture with tumor-derived MDSCs; then, the expression of GAS5 was detected by qRT-PCR. (**R**,**S**) STAT3-depleted MDA-MB-231cells were co-cultured with or without MDSCs from primary TNBC tissues; the proteins of interest were detected with Western blotting (**R**), and the GAS5 level was measured by qRT-PCR (**S**). **, *p* < 0.01; ***, *p* < 0.001.

**Figure 6:**
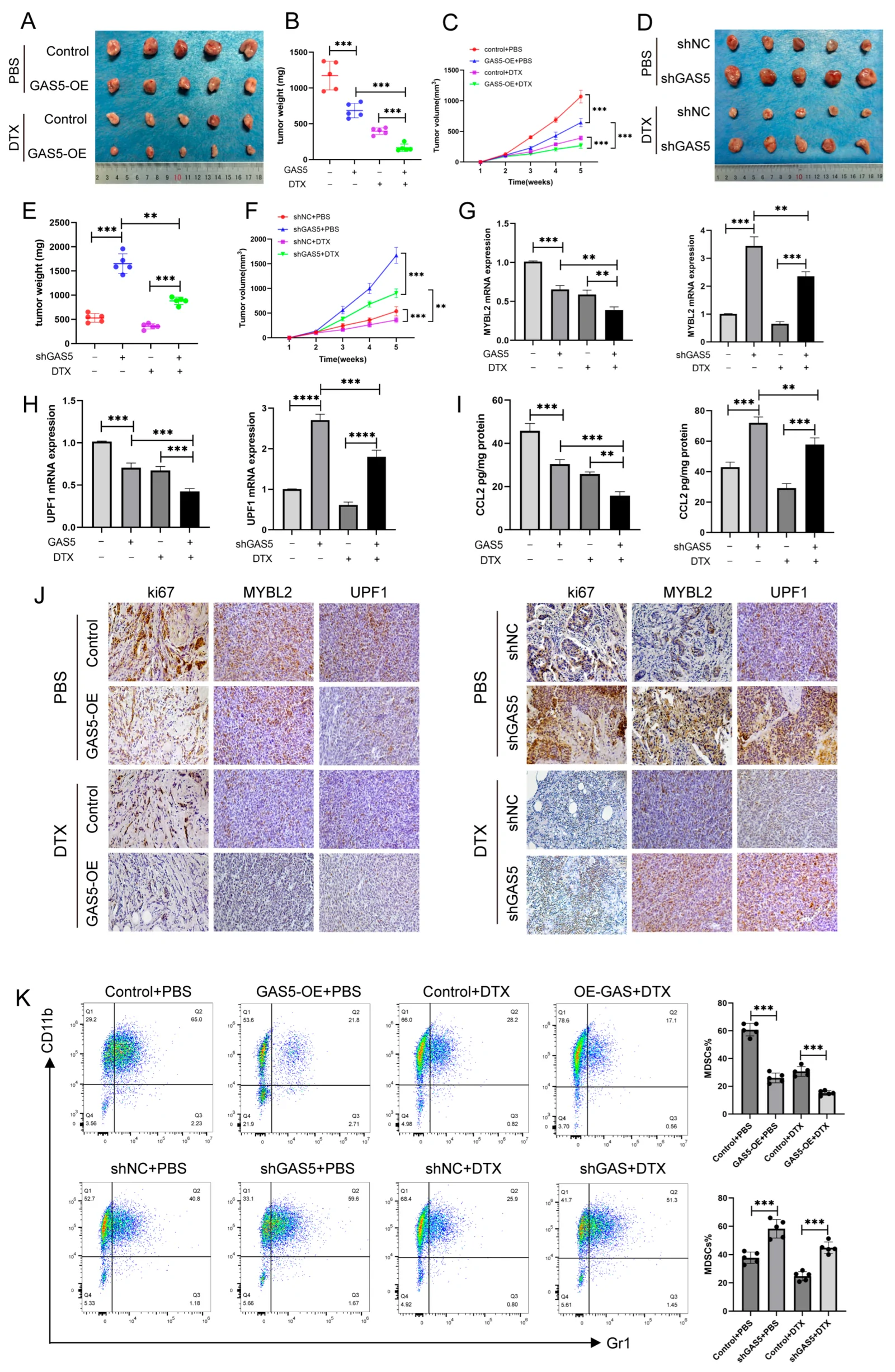
Overexpression of GAS5 suppressed the growth, DTX resistance, and MDSC recruitment in TNBC in vivo. MDA-MB-231 cells with GAS5-OE, shGAS5, or control vector were injected subcutaneously into BALB/c nude mice. Then, the mice were treated with PBS or docetaxel (1 mg/kg) for 4 weeks. (**A**,**D**) Representative images of tumors excised from xenografts (*n* = 5). (**B**,**E**) Tumor weights were measured after sacrifice of the mice. (**C**,**F**) The volumes of the xenografts were monitored weekly. (**G**,**H**) The expression of MYBL2 (**G**) and UPF1 (**H**) in tumor tissues was detected by qRT-PCR. (**I**) The level of CCL2 in tumor tissue lysates was quantified by ELISA. (**J**) Representative images of IHC staining of Ki67, MYBL2, and UPF1 in the tumor tissues. Scale bar = 100 μm. (**K**) Flow cytometry analysis of MDSCs in tumor tissues. ** *p* < 0.01; *** *p* < 0.001.

## Data Availability

All data generated in this study are available from the corresponding author upon reasonable request.

## Supplementary Materials

The following supporting information can be downloaded at: https://www.mdpi.com/article/10.3390/cancers18193133/s1, Figure S1: Functional validation of immunosuppressive activity in isolated primary TNBC tissues-derived MDSCs subsets; Figure S2: Colony formation assays of MDA-MB-231 and HCC1937 cells were performed to evaluate the effect of GAS5 overexpression on cell proliferative ability; Figure S3: GAS5 overexpression suppresses MDSC chemotaxis toward TNBC-conditioned medium. Table S1: Primer sets used for qRT-PCR; Table S2: The sequences of siRNAs; Table S3: The antibodies used in this study.
