# Supplementary material for: GAS5 Downregulation Promotes the Progression of Triple-Negative Breast Cancer by Regulating Tumor Cells-MDSCs Crosstalk
Source: Cancers (Basel). 2026 Sep 27;18(19):3133. doi: 10.3390/cancers18193133 (PMC13663935; doi:10.3390/cancers18193133)
Supplement: Supplementary file 1 [file cancers-18-03133-s001.zip › Supplementary figures.pdf]

Supplementary figure

Supplementary figure S1

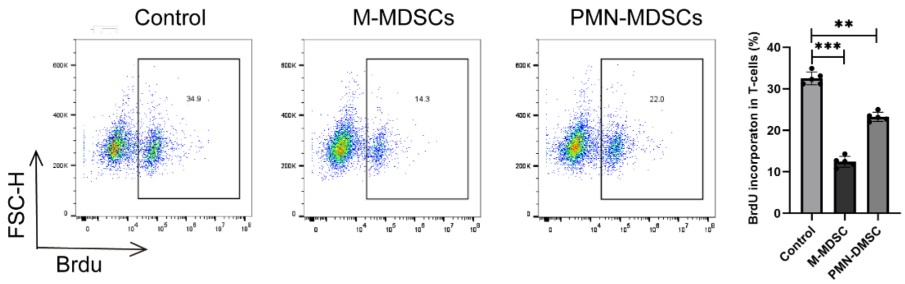

Supplementary Figure S1. Functional validation of immunosuppressive activity in isolated primary TNBC tissues-derived MDSCs subsets. (A) Representative flow cytometry histograms showing BrdU dilution in anti-CD3/CD28-stimulated $CD3^{+}$T cells co-cultured with M-MDSCs, PMN-MDSCs, or without MDSCs (T cell only). The unstimulated T-cell group served as a negative control. (B) Quantification of the T-cell proliferation across different co-culture conditions. Data are presented as mean±SD (n = 5 independent donors). **, $P < 0.01$; ***, $P < 0.001$

Supplementary figure S2

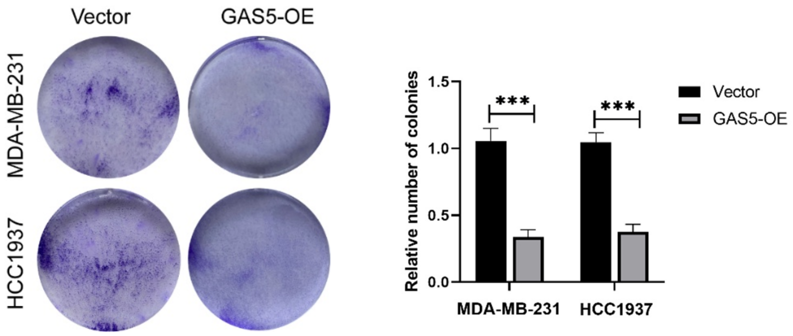

Supplementary figure S2. Colony formation assays of MDA-MB-231 and HCC1937 cells were performed to evaluate the effect of GAS5 overexpression on cell proliferative ability. Scale bar =500μm. MDA-MB-231 and HCC1937 cells stably transduced with either the empty vector (Vector) or the GAS5 overexpression lentivirus (GAS5-OE) were harvested during the logarithmic growth phase. Single-cell suspensions were prepared, and viable cells were counted using a hemocytometer with Trypan blue exclusion. Cells were seeded in 6-well plates at a density of 500 cells per well (in 2 mL of complete DMEM containing 10% FBS) and cultured in a humidified incubator at 37 °C with 5% CO2 for 10–14 days. The medium was replaced every 3 days to ensure optimal growth conditions. Surviving colonies (50 cells per colony) were visualized with 0.1% crystal violet (Sigma-Aldrich) staining. Images of the entire wells were captured using a digital camera.

Supplementary figure S3

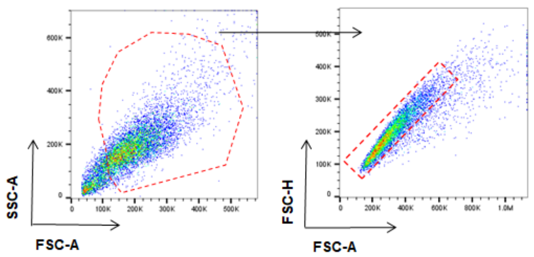

Supplementary figure S3. GAS5 overexpression suppresses MDSC chemotaxis toward TNBC-conditioned medium. Representative flow cytometry dot plots showing the gating strategy for migrated MDSCs in the lower chamber of transwell plates.
