# Supplementary material for: GAS5 Downregulation Promotes the Progression of Triple-Negative Breast Cancer by Regulating Tumor Cells-MDSCs Crosstalk
Source: Cancers (Basel). 2026 Sep 27;18(19):3133. doi: 10.3390/cancers18193133 (PMC13663935; doi:10.3390/cancers18193133)
Supplement: Supplementary file 1 [file cancers-18-03133-s001.zip › Supplementary tables.pdf]

Supplementary Table S1

Table S1. Primer sets used for qRT-PCR

Primer set	Primers	Sequence (5'-3')
GAS5	Forward	ACTCCTGTGAGGTATGGTGC
	Reverse	CCATCATCTCCTTTGTCACCAGG
MYBL2	Forward	CCGGAGCAGAGGGATAGCA
	Reverse	CAGTGCGGTTAGGGAAGTGG
YTHDF2	Forward	GCAAGCAATGTTCCAAAAG
	Reverse	GCAATATCAGCCCAAGATG
CCL2	Forward	AGAATCACCAGCAGCAAGTGTCC
	Reverse	TCCTGAACCCACTTCTGCTTGG
UPF1	Forward	ACCACGAAGTTGCTGAAGG
	Reverse	ACACAGGACAGGATGATGAAG
IL-6	Forward	AGACAGCCACTCACCTCTTCAG
	Reverse	TTCTGCCAGTGCCTCTTTGCTG
β-actin	Forward	GGGAAATCGTGCGTGACATT
	Reverse	GGAACCGCTCATTGCCAAT
musMYBL2	Forward	GACGCGCTGCGAGGAT
	Reverse	GTCTCTCTGCTCCAGGAGGT
musUPF1	Forward	CATCAGGGCATTGGGTTCCT
	Reverse	ACCCACAATGATCACGCCAT
musβ-actin	Forward	CATTGCTGACAGGATGCAGAAGG
	Reverse	CATTGCTGACAGGATGCAGAAGG

Supplementary Table S2

Table S2. The sequences of siRNAs

siRNAs		Sequence (5'-3')
siYTHDF2 #1	Sense	CCACAGGCAAGGCCCAAUAAU
	Antisense	AUUAUUGGGCCUUGCCUGUGG
siYTHDF2 #2	Sense	UCUGGAUAUAGUAGCAAUUAU
	Antisense	AUAAUUGCUACUAUAUCCAGA
siYTHDF2 #3	Sense	CUAGAGAACAACGAGAAUAAA
	Antisense	UUUAUUCUCGUUGUUCUCUAG
siMYBL2 #1	Sense	UUUUGAUCUUCCUCUUUGGUC
	Antisense	CCAAAGAGGAAGAUCAAAAGG
siMYBL2 #2	Sense	ACUCAAUGACCUUUUGAUCUU
	Antisense	GAUCAAAAGGUCAUUGAGUUG
siMYBL2 #3	Sense	AUUCUUCACAGCAUUGUCCGU
	Antisense	GGACAAUGCUGUGAAGAAUCA
siUPF1 #1	Sense	AGUAUGUGUCCUCUUCAUCUU

	Antisense	GAUGAAGAGGACACAUACUAC
siUPF1 #2	Sense	UGGUAUUACAGUAAACCACGC
	Antisense	GUGGUUUACUGUAAUACCAGC
siUPF1 #3	Sense	AACUUCUUGUCAUAGUCAGCC
	Antisense	CUGACUAUGACAAGAAGUUGA
siUPF1 #1	Sense	UUCUAUUCUAGCACAUUGCGC
	Antisense	GCAAUGUGCUAGAAUAGAAGA
siUPF1 #2	Sense	UUAUUCAAACCAUUAUUUGUA
	Antisense	CAAAUAAUGGUUUGAAUAAAG
siUPF1 #3	Sense	UUAAUACCUUUCUUUAUUCAA
	Antisense	GAAUAAAGAAAGGUAUUAAUG

Supplementary Table S3

Table S3. The antibodies used in this study

**Antibody**	**Source (Catalog No.)**
MYBL2 Rabbit Polyclonal antibody	Proteintech (18896-1-AP)
UPF1 Mouse Monoclonal antibody	Proteintech (66898-1-Ig)
CCL2 Rabbit Polyclonal antibody	Proteintech (29547-1-AP)
STAT3 Rabbit Polyclonal antibody	Proteintech (10253-2-AP)
p-STAT3 Mouse Monoclonal antibody	Proteintech (60479-1-Ig)
YTHDF2 Rabbit Polyclonal antibody	Proteintech (24744-1-AP)
MYBL2 Rabbit Monoclonal Antibody	CST(33056)
Ki67 Rabbit Polyclonal antibody	Abcam (ab15580)
β-Actin Mouse Monoclonal antibody	Proteintech (66009-1-Ig)
PerCP-Cyanine5.5 Anti-mouse/human CD11b	BioLegend (101227)
BV 650 Anti-mouse Ly-6G/Ly-6C (Gr-1)	BioLegend (108441)
APC Anti-human CD11b	BioLegend (301310)
PE Anti-human CD33	BD Biosciences (555450)
PerCP-Cy5.5 Anti-human HLA-DR	BioLegend (307628)
FITC Anti-human CD14	BD Biosciences (555397)
PE-Cy7 Anti-human CD15	BioLegend (301908)
BV421 Anti-human CD3	BioLegend (317344)
Anti-human CD28	BioLegend (302942)
